# Supplementary material for: Developing a quality indicator system for evaluating internet plus home care nursing services based on the SERVQUAL model: a Delphi-analytic hierarchy process study
Source: PeerJ. 2024 Nov 12;12:e18281. doi: 10.7717/peerj.18281 (PMC11566512; doi:10.7717/peerj.18281)
Supplement: Supplemental Information 1 — The detailed general information of the consulted experts, the comparison of degree of authority and the coordination of experts’ opinions in the two-round delphi consultation. [file peerj-12-18281-s001.docx]

# Developing a Quality Indicator System for Evaluating Internet Plus Home Care Nursing Services Based on the SERVQUAL Model：a Delphi-Analytic Hierarchy Process Study

Lei YE^1^, Shulan YANG^1^, Biyan JIANG^1^, Caixia LIU^1^, Xiaoqing JIN^1^, Polun CHANG^2^,

^1^ Department of Nursing, Zhejiang Hospital, Hangzhou, China

^2^ Institute of Biomedical Informatics, Yang-Ming Chiao-Tung University, Taiwan

Corresponding Author:

Shulan YANG^1^, MSc

Department of Nursing, Zhejiang Hospital, Hangzhou, China，310000

Email address: 947373396@qq.com

Table 1. General information of the experts (n=17).

| Item | N | Proportion |
| --- | --- | --- |
|  |  |  |
| **age (year)** |  |  |
| 30-39 | 2 | 11.76% |
| >=40 | 15 | 88.24% |
| **working years** |  |  |
| 11-20 | 2 | 11.76% |
| ≥20 | 15 | 88.24% |
| **education background** |  |  |
| Bachelor | 9 | 52.94% |
| Master | 4 | 23.53% |
| Doctor | 4 | 23.53% |
| **professional titles** |  |  |
| Medium-grade | 3 | 17.65% |
| Senior-grade | 14 | 82.35% |
| **specialized fields** |  |  |
| Nursing education | 4 | 23.53% |
| Clinical nursing | 5 | 29.41% |
| Health management | 5 | 29.41% |
| Health Informatics | 3 | 17.65% |

Table 2. The degree of expert authority in two rounds.

| **Round** | **Judgement coefficient (Ca)** | **Familiarity coefficient (Cs)** | **Authority coefficient (Cr)** |
| --- | --- | --- | --- |
|  |  |  |  |
| Delphi Round 1 | 0.967 | 0.857 | 0.912 |
| Delphi Round 2 | 0.925 | 0.925 | 0.925 |

Table 3. The coordination degree of expert opinions.

| **Item** | **Delphi Round 1** | | | **Delphi Round 2** | | |
| --- | --- | --- | --- | --- | --- | --- |
|  |  | | |  | | |
|  | Kendall’s W | *X*^2^ | *P* | Kendall’s W | *X*^2^ | *P* |
|  |  |  |  |  |  |  |
| primary indicator | 0.337 | 22.906 | <0.001 | 0.232 | 14.839 | <0.001 |
| secondary indicator | 0.256 | 60.873 | <0.001 | 0.280 | 62.815 | <0.001 |
| total | 0.262 | 84.565 | <0.001 | 0.265 | 80.514 | <0.001 |

Table 4**.** Results of expert correspondence on primary indicators in Delphi round 1 and 2.

| **Primary Indicator** | **Delphi Round 1** | | **Delphi Round 2** | |
| --- | --- | --- | --- | --- |
|  | **Importance**  **（x±s）** | **CV** | **Importance**  **（x±s）** | **CV** |
|  |  |  |  |  |
| **reliability** | 4.938±0.25 | 0.051 | 4.941±0.243 | 0.049 |
| **assurance** | 4.813±0.403 | 0.084 | 4.824±0.393 | 0.081 |
| **tangibles** | 4.5±0.516 | 0.115 | 4.529±0.514 | 0.114 |
| **empathy** | 4.438±0.512 | 0.115 | 4.471±0.514 | 0.115 |
| **responsiveness** | 4.75±0.447 | 0.094 | 4.765±0.437 | 0.092 |

Table 5. Results of expert correspondence on secondary indicators in Delphi round 1 and 2.

| **Primary indicator** | **Secondary indicator** | **Delphi Round 1** | | **Delphi Round 2** | |
| --- | --- | --- | --- | --- | --- |
|  |  | **Importance**  **（x±s）** | **CV** | **Importance**  **（x±s）** | **CV** |
|  |  |  |  |  |  |
| **reliability** | Rational of the service plan | 4.813±0.403 | 0.084 | 4.824±0.393 | 0.081 |
|  | Guarantee of the service execution | 4.875±0.342 | 0.070 | 4.882±0.332 | 0.068 |
|  | Proper presentation of the service records and feedback | 4.375±0.5 | 0.114 | 4.412±0.507 | 0.115 |
| **assurance** | Qualification of the agent | 4.875±0.342 | 0.070 | 4.882±0.332 | 0.068 |
|  | Knowledge and skill of the provider | 4.875±0.342 | 0.070 | 4.882±0.332 | 0.068 |
|  | Attitude of the provider | 4.6±0.507 | 0.110 | 4.625±0.5 | 0.108 |
|  | The ability of provide on-site support | 4.867±0.352 | 0.072 | 4.875±0.342 | 0.070 |
| **tangibles** | The degree of platform support | 4.625±0.5 | 0.108 | 4.647±0.493 | 0.106 |
|  | The intensity of platform attention | 4.375±0.619 | 0.142 | 4.412±0.618 | 0.140 |
|  | The level of homogenization among providers | 4.313±0.602 | 0.140 | 4.353±0.606 | 0.139 |
|  | The consistency of service and demand | 4.938±0.25 | 0.051 | 4.941±0.243 | 0.049 |
| **empathy** | The ability to provide personalized services | 4.533±0.516 | 0.114 | 4.563±0.512 | 0.112 |
|  | Give priority to the interests of the clients | 4.467±0.743 | 0.166 | 4.375±0.806 | 0.184 |
| **responsiveness** | Responsiveness of routine service requirements | 4.688±0.479 | 0.102 | 4.706±0.47 | 0.100 |
|  | Active response to temporary service requirements | 4.188±0.75 | 0.179 | 4.875±0.342 | 0.174 |
