# Supplementary material for: Developing a quality indicator system for evaluating internet plus home care nursing services based on the SERVQUAL model: a Delphi-analytic hierarchy process study
Source: PeerJ. 2024 Nov 12;12:e18281. doi: 10.7717/peerj.18281 (PMC11566512; doi:10.7717/peerj.18281)
Supplement: Supplemental Information 2 [file peerj-12-18281-s002.docx]

**Evaluation of Internet + home care services based on SERVQUAL model**

**Scale index was constructed by inquiry questionnaire**

**Expert letter inquiry questionnaire (first round)**

Dear experts:

shalom! Invite you to attend our letter inquiry, thank you for taking time out of your busy schedule to fill in our letter inquiry form!

What we are studying is the Internet + Home Care Service Quality Evaluation Scale index constructed based on the SERVQUAL model, so as to compile the Internet + Home Care Service Quality Questionnaire to improve the service quality.

Given your academic attainments and management experience, invited you to fill in this questionnaire, please fill in detailed order, your opinion will be as we build Internet + home care service quality assessment scale index of important theoretical basis, the questionnaire content is limited to this study, not for other purposes, we will do the survey content strictly confidential.

This questionnaire is the first round of Delphi expert letter inquiry questionnaire of "Internet + Home Care Service Quality Evaluation Scale based on SERVQUAL model". Complete this expert letter inquiry needs to occupy some of your valuable time. Thank you very much for your understanding, support and cooperation! Due to the time, I sincerely hope that you can return the questionnaire within a week. Tel.: 13606542154, email address: 375857761@qq.com 。We would be very grateful. I wish you good health and a smooth work!

| You are welcome to fill out the e-form | 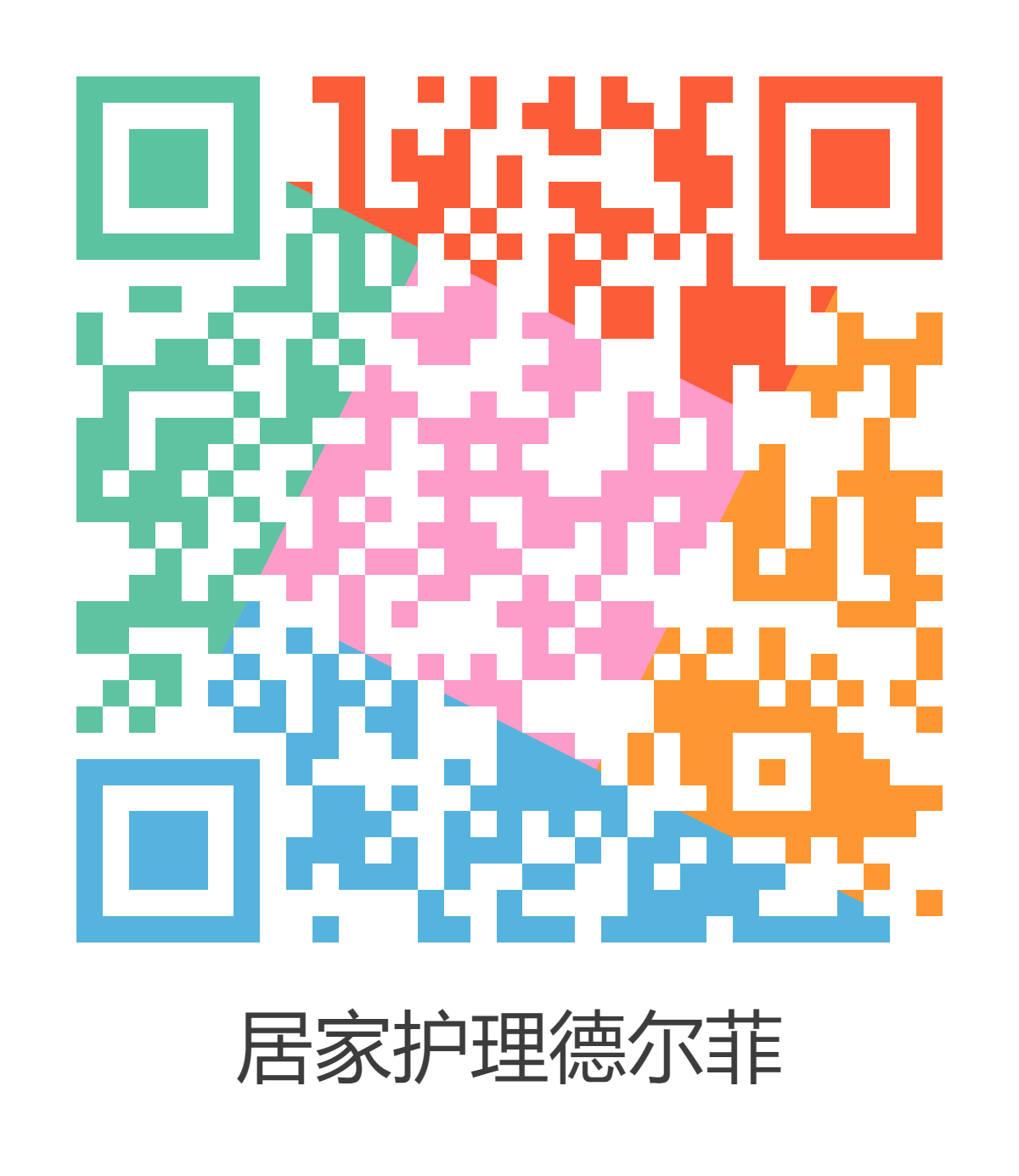 |
| --- | --- |

Fill in the form:

**1. This letter inquiry form is the first round of Delphi expert letter inquiry questionnaire, which is divided into three parts. The first part is a research introduction; the second part is formal letter content; the third part is the basic information sheet of experts.**

2. In the second part, we divide the indicators into first-level indicators and second-level indicators. When you fill in the form, please judge whether each index item belongs to the corresponding classification, and make a choice according to the order of "5 is very important, 4 important, 3 is generally important, 2 is not very important, 1 is not important". If you have other suggestions, please fill in "Do you need to add or remove an indicator".

3. In the third part, you can directly fill in the corresponding column. **Part I, Study Introduction**

China has officially entered the aging society, and the demand for elderly care services is also increasing. The increase of the elderly population means that higher requirements for medical services should be set. However, there is still a shortage and imbalance of medical resources in China. The traditional medical service model has failed to meet the demand for mass medical services, and the demand for home care services has increased sharply.

"Internet + home care service" as a new medical service model is in the ascendant. The purpose of this study is to develop the scale index suitable for evaluating the Internet + Home Care Service Quality Evaluation, and to compile the Internet + Home Care Service Quality Questionnaire.

SERVQUAL Model is a tool to measure the quality of service, its theoretical core is "service quality gap model", namely the quality of service depends on the difference between the service level (also known as "expectation-perception" model), the user's expectation is the prerequisite for quality service, the key to provide quality service is to exceed the user's expectations. For each index in the evaluation scale, two scores (1-7 points) are given for the service expectation value and service expectation value and service perception value. The "SERVQUAL score = feeling service score" is calculated to evaluate the service quality. A positive SERVQUAL score indicates good service quality; a positive SERVQUAL score of 0 indicates satisfactory general service quality; and a negative SERVQUAL score indicates poor service quality.

Application of this scale is "Internet + home care" users, on the basis of SERVQUAL, from the perspective of the user, the service quality evaluation, through five dimensions (tangible, reliability, responsiveness, guarantee, empathy) study "Internet + home care" service quality, understand the user demand for service, improve service quality, increase the competitiveness of similar services, to provide a better level of home endowment service. The evaluation model, as an important theoretical basis for constructing the index of Internet + home care service quality evaluation scale, will evaluate the service quality. In the early stage of the study, the research group conducted literature search and semi-structure interview, and proposed 5 first-level indicators (five dimensions of SERVQUAL model) and 15 second-level indicators (selected from 22 indicators related to SERVQUAL model).

**Primary indicator 1: Tangibles**

**Level 1 indicator 2: Reliability**

**Level 1 index 3: Responsiveness**

**Level 1 index 4: Assurance**

**Level 1 index 5: Empathy**

**The second part of the formal letter inquiry content**

**Internet + Home Care Service quality evaluation scale index based on SERVQUAL model**

The contents of the letter inquiry include two tables, namely Table 1 letter inquiry scores and opinions on first level indicators, and Table 2 letter inquiry scores and opinions on second level indicators.

Table 1 Letter inquiry scores and opinions on first-level indicators

🗸Fill in the form: Please score the importance of the first-level index items (mark "" in the scoring box or the number); but also hope that you will be able to modify or add comments on the "expression of the first-level index item", "description of the first-level index item" and "whether to add or delete a level index".

| **Level 1 index entry** | | **Importance score of the first-level index entry** | | | | | **Modification of the level 1 indicators**  **And supplementary opinions** |
| --- | --- | --- | --- | --- | --- | --- | --- |
| Level 1 index entry | explanation | 5 Points  (very important) | 4 Points  (important) | Three points  (Generally important) | Two points  (Not too important) | 1 Points  (Very unimportant) |  |
| **1 Tangibles** | Service-related facilities and circumstances are directly perceived by service users; for example, the information platform, service provider’s personal image, and performance. |  |  |  |  |  |  |
| **2 Reliability** | The ability of the Internet+ HCN service to provide reliable services, which accurately meet user’s service demand, and the ability to guarantee service execution. |  |  |  |  |  |  |
| **3 Responsiveness** | The ability of responding to users’ service demands. |  |  |  |  |  |  |
| **4 Assurance** | The relevant ability of the platform, agent, and provider to gain trust from the service user; for example, provider’s knowledge, skill, and attitude. |  |  |  |  |  |  |
| **5 Empathy** | The ability of the platform, agent, and provider to attract the adhesiveness of the users. |  |  |  |  |  |  |
| You suggest  Add or remove  Level I entries |  | | | | | | |

Table 2 Inquiry scores and opinions on secondary indicators

🗸Fill in the form: Please score the appropriateness of the secondary index entry under each primary index item (mark "" in the corresponding scoring box or fill in the number); and also hope that you can modify or add comments on the "expression of the secondary index entry" and "whether to delete or add a secondary index entry".

| **Level 1 index entry** | **Secondary index entry** | **explanation** | **Importance score of the secondary index entries** | | | | | **Modification of the secondary index items**  **And supplementary opinions** |
| --- | --- | --- | --- | --- | --- | --- | --- | --- |
|  |  |  | 5 Points  (very important) | 4 Points  (important) | Three points  (Generally important) | Two points  (Not too important) | 1 Points  (Very unimportant) |  |
| **1 Tangibles** | ① Degree of platform support | The degree of platform support in view of the user. |  |  |  |  |  |  |
|  | ② Intensity of platform attention | The intensity of platform attention in view of the user. |  |  |  |  |  |  |
|  | ③ Level of homogenization among providers | The level of homogenization among providers in view of the user, such as wearing a unified professional uniform and following the same operating procedures. |  |  |  |  |  |  |
|  | ④ Consistency of service and demand | The consistency of service and demand in view of the user. |  |  |  |  |  |  |
| **2 Reliability** | ⑤ Rational of the service plan | Once ordered, a correct service plan is activated. The assigned HCN service provider is qualified with a proper professional certification that is in line with the active service plan. |  |  |  |  |  |  |
|  | ⑥ Guaranteed service execution | The implementation of the HCN service is consistent with its description. In case of emergency, the assigned HCN service provider could offer emergency advice, and perform necessary and prompt rescue or transfer measures. |  |  |  |  |  |  |
|  | ⑦ Proper presentation of the service records and feedback | Once complete, the service records and feedback are properly presented. |  |  |  |  |  |  |
| **3 Responsiveness** | ⑧ Responsiveness of regular service requirements | The ability to respond to regular and routine service requirements. |  |  |  |  |  |  |
|  | ⑨ Active response to temporary service requirements | The ability to actively respond to temporary service requirements. |  |  |  |  |  |  |

| **Level 1 index entry** | **Secondary index entry** | **explanation** | **Importance score of the secondary index entries** | | | | | **Modification of the secondary index items**  **And supplementary opinions** |
| --- | --- | --- | --- | --- | --- | --- | --- | --- |
|  |  |  | 5 Points  (very important) | 4 Points  (important) | Three points  (Generally important) | Two points  (Not too important) | 1 Points  (Very unimportant) |  |
| **4 Assurance** | ⑩ Qualification of the agent and the service provider | Qualification of the agent and staffs are indicated in the platform. |  |  |  |  |  |  |
|  | ⑪ Knowledge and skill of the service provider | The assigned HCN service provider is proficient in the knowledge and skill required by the service task. |  |  |  |  |  |  |
|  | ⑫ Attitude of the service provider | The assigned HCN service provider performs professional communication. |  |  |  |  |  |  |
|  | ⑬ Ability to provide on-site support | In case of emergency, the assigned HCN service provider could seek help through the platform. |  |  |  |  |  |  |
| **5 Empathy** | ⑭ Ability to provide personalized services | The ability to provide personalized services in view of the user. |  |  |  |  |  |  |
|  | ⑮ Give priority to the interests of the clients | The ability to give priority to the interests of the clients in view of the user. |  |  |  |  |  |  |
| You suggest  Additional or deleted secondary entries |  | | | | | | | |

**The third part of the expert basic situation investigation and self-evaluation form**

Fill in the form description: please fill in or check the corresponding items according to the actual situation (all your information will be kept strictly confidential).

Table 4. Basic information of the experts

| **surname and personal name** |  |
| --- | --- |
| **sex** |  |
| **age** |  |
| **work unit** |  |
| **administrative or technical offices** |  |
| **post** |  |
| **professional ranks and titles** |  |
| **record of formal schooling** |  |
| **Work field** | domain |
| **working life** | year |
| **Study / field of familiarity and years** | domain |
|  | year |

Table 5. Self-evaluation table of expert authority degree

| In the letter inquiry, the following four judgments are based on the degree of influence on you | | | | | | | |
| --- | --- | --- | --- | --- | --- | --- | --- |
| Influence degree  Judgment basis | | big | | centre | | small | |
| 1. **hands-on** | |  | |  | |  | |
| 1. **theoretical analysis** | |  | |  | |  | |
| 1. **The peer understand**   (For example, refer to relevant domestic information) | |  | |  | |  | |
| 1. **Intuitive choice** | |  | |  | |  | |
| Your familiarity with Internet + Home care related content | | | | | | | |
| **know...well** | **More familiar with** | | **Generally familiar with** | | **Not familiar with** | | **Not familiar with** |
|  |  | |  | |  | |  |

The above are all the contents of this letter of inquiry. Thank you for your support and cooperation with this study. Wish you a happy life!
