# Supplementary material for: Developing a quality indicator system for evaluating internet plus home care nursing services based on the SERVQUAL model: a Delphi-analytic hierarchy process study
Source: PeerJ. 2024 Nov 12;12:e18281. doi: 10.7717/peerj.18281 (PMC11566512; doi:10.7717/peerj.18281)
Supplement: Supplemental Information 4 [file peerj-12-18281-s004.docx]

**Interview Guide（For Service User）**

**Introduction**

1. Self-introduction: Hello, I am XXX from the XXX research team. We are currently conducting a research on "Internet + Home Care Nursing Services". Today, I would like to have a brief interview with you to get some valuable opinions and suggestions from you.

**Interview**

1. Please briefly introduce yourself, including your age, gender, occupation, family situation, etc.

2. Have you ever used Internet + Home Care Nursing Services? If you have, please describe your experience and feelings of using the service. If you haven't, what do you think is the reason why you haven't chosen to use the service?

3. In your opinion, what are the advantages and disadvantages of Internet + Home Care Nursing Services? What kind of service can best meet your needs?

4. How do you think the quality of Internet + Home Care Nursing Services is? Have you ever encountered a situation where you were not satisfied with the service? If so, please describe the specific situation and how you handled it. The interviewer can continue to ask questions based on the 5-dimension RATER model, a simplified version of the SERVQUAL model, which is composed of reliability, assurance, tangibles, empathy, and responsiveness.

5. Do you think the price of Internet + Home Care Nursing Services is reasonable? Are you willing to pay more for the service? Why?

6. What abilities and qualities should the provider of Internet + Home Care Nursing Services have? What aspects do you value the most?

7. What do you think the government and society should do to standardize Internet + Home Care Nursing Services? What problems do you hope Internet + Home Care Nursing Services can solve for you?

**Ending**

1. Thank you very much for taking the time to accept our interview. Your opinions and suggestions are very important to us, and we will carefully sort out and analyze your answers.

**Interview Guide （For Service Provider）**

**Introduction**

1. Self-introduction: Hello, I am XXX from the XXX research team. We are currently conducting a research on "Internet + Home Care Nursing Services". Today, I would like to have a brief interview with you to get some valuable opinions and suggestions from you.

**Interview**

1. Please briefly introduce yourself, including your age, gender, occupation, work experience, etc.

2. Have you ever provide Internet + Home Care Nursing Services? If you have, please describe your experience and feelings of providing the service. If you haven't, what do you think is the reason why you haven't want to join the service?

3. In your opinion, what are the advantages and disadvantages of Internet + Home Care Nursing Services?

4. How do you think the quality of Internet + Home Care Nursing Services is? Have you ever encountered a situation where you were not satisfied with the service platform? If so, please describe the specific situation and how you handled it. The interviewer can continue to ask questions based on the 5-dimension RATER model, a simplified version of the SERVQUAL model, which is composed of reliability, assurance, tangibles, empathy, and responsiveness.

5. Do you think the earnings of Internet + Home Care Nursing Services are attractive? Are you willing to earn extra gains from the service? Why?

6. What abilities and qualities should the provider of Internet + Home Care Nursing Services have? What aspects do you value the most?

7. What do you think the government and society should do to standardize Internet + Home Care Nursing Services?

**Ending**

1. Thank you very much for taking the time to accept our interview. Your opinions and suggestions are very important to us, and we will carefully sort out and analyze your answers.
